# The work–life balance and psychosocial well-being of South Korean workers

**DOI:** 10.1186/s40557-018-0250-z

**Published:** 2018-06-05

**Authors:** Jae Won Yang, Chunhui Suh, Chae Kwan Lee, Byung Chul Son

**Affiliations:** Department of Occupational and Environmental Medicine & Institute of Environmental and Occupational Medicine, Pusan Paik Hospital, Inje University, Hospital, 75, Bokji-ro, Busanjin-gu, Busan, 47392 Republic of Korea

**Keywords:** Work–life balance, Psychosocial well-being, KWCS, WHO-5 index

## Abstract

**Background:**

It is challenging to balance work and life, and little attention has been paid to the work–life balance and psychosocial well-being of South Koreans. We assessed the association between work–life balance and psychosocial well-being among paid Korean workers.

**Methods:**

This study was based on data from the fourth Korean Working Conditions Survey. We evaluated only paid workers, which constituted 30,649 of the total of 50,007 subjects surveyed. Poor work–life balance was defined based on the goodness of fit between working hours and social commitments. Well-being was measured using the World Health Organization WHO-5 index. Poisson regression with robust variances was used to calculate the estimated prevalence ratios (PRs) with confidence intervals.

**Results:**

Poor work–life balance was associated with poor psychosocial well-being (PR = 1.25; 95% CI 1.21 to 1.28) even after adjusting for work-related and individual characteristics. Poor well-being was associated with low-level job autonomy (PR = 1.06; 95% CI 1.03 to 1.09), working for ≥53 h per week (PR = 1.10; 95% CI 1.06 to 1.14), blue-collar status (PR = 1.16; 95% CI 1.11 to 1.21), low-level support at work (PR = 1.32; 95% CI 1.29 to 1.36), age ≥ 50 years (PR = 1.21; 95% CI 1.15 to 1.26), the female gender (95% CI PR = 1.04; 95% CI 1.01 to 1.07), and cohabitation (living with somebody) (PR = 1.08; 95% CI 1.04 to 1.12). Good well-being was associated with high-intensity work (PR = 0.96; 95% CI 0.94 to 0.99), being the secondary earner in a household (PR = 0.82; 95% CI 0.79 to 0.85), and higher income (PR = 0.75; 95% CI 0.71 to 0.79).

**Conclusion:**

Work–life balance was associated with psychosocial well-being after adjusting for both work-related and individual characteristics.

## Background

Successfully reconciling work and non-work time is challenging regardless of life stage or profession [1]. Both demographic features and the work-related environment have changed in recent times. The traditional sole male breadwinner is less common today. Women work and dual-earning couples are common. The lack of single-parent housing combined with increasing work demands and rapid industrial changes render it harder for workers to balance work with life; stress levels are increasing [2, 3]. It is important to define an appropriate work–life balance and to understand the current situation in South Korea.

No widely accepted definition of work–life balance is yet available; the idea is complex. Suggested definitions include “equilibrium or an overall sense of harmony in work and private life” [4], “an adequate amount of resources to respond effectively to the demands of their and family roles” [5], and “balancing the life demands of various life-roles” [6]. Harmonization of life and work improves mental and physical health. A good balance between work and life improves job satisfaction, psychosocial well-being, and the overall quality-of-life [7]. When work and personal life are poorly balanced the consequences include decreased job satisfaction, poor psychosocial well-being, and a lower quality-of-life [8–10]. Failure to achieve the required balance impairs mental health, and triggers burnout, depression, and family conflict [11, 12].

The Organization for Economic Cooperation and Development (OECD) ranked South Korea 36th among 38 OECD countries in terms of work–life balance, because of very long working hours, gender inequality, and insufficient time for leisure and personal care. The longer the work hours, the less time is available to spend with others, to engage in leisure activities, and to eat and sleep. Overall well-being is compromised, as are physical and mental health http://www.oecdbetterlifeindex.org/topics/work-life-balance/. Of all Korean workers, 23.1% work ≥50 h/week, compared to the OECD average of 13%. The OECD average for work hours per week is 37.6 h; the South Korean average is 44.3 h [13].

A perceived good balance between work and personal life predicts psychosocial well-being [14]. The World Health Organization questionnaire WHO-5 can be used to evaluate the “state of well-being” and is a useful measure of subjective physical, mental, and social health [15]. The tool also assesses the overall quality-of-life, emotional state, and depression [16]. Impaired psychological well-being may reduce job involvement and increase absenteeism [17]. Individuals with greater psychosocial well-being are more dedicated to work, and more productive and happier, than others [18].

As mentioned above, an imbalance between work and private life is of growing concern in South Korea. However, the issue has been little-studied. Our purpose was to identify associations between work–life balance and psychosocial well-being using data from the fourth Korean Working Conditions Survey.

## Methods

### Data and study samples

This study was based on the fourth Korean Working Conditions Survey (KWCS 2014) performed by the Korea Occupational Safety and Health Agency. The basic sample design is multistage random sampling. The enumeration districts in the 2010 Population and Housing Census were used for sampling. Data were gathered via face-to-face interviews in homes, using the questionnaire. The survey gathered comprehensive information on working conditions to define workforce changes and the quality of work and life. The survey was performed in 2014 and targeted the economically active population aged ≥15 years who were paid workers or self-employed at the time of interview. The survey data were weighted with reference to the economically active population, in that the sample distributions by region, locality, sex, age, economic activity, and occupation were identical to those of the overall economically active population at the time of the survey. We restricted our analysis to paid workers. Therefore, we included only 30,649 of a total of 50,007 workers. Military personnel and those who did not respond were also excluded [19].

### Measures

Psychosocial well-being, our outcome of interest, was measured using five items of the WHO-5 scale: “I feel cheerful and in good spirits/I feel calm and relaxed/I feel active and vigorous/I wake up feeling fresh and rested/My daily life is filled with things that interest me”. Each item is scored from 0 to 5. A raw score (0–25) is calculated by totaling the Figs. A score < 13 indicates poor wellbeing. We dichotomized the scores into good and poor well-being [20].

Work–life balance, our primary exposure of interest, was assessed by a single question: “In general, do your working hours fit in with your family or social commitments outside work?”. The answers were dichotomized as good (“very well” or “well”) and poor (“not very well” or “not at all well”). Other covariates were divided into two categories: Individual and work-related. Of many possible work-related characteristics, we explored job type, weekly work hours, work intensity, job autonomy, job insecurity, and support at work. Job type was categorized as white-collar (managers, professionals, and technicians), service and sales, and blue-collar (agriculture/fishery workers, and skilled workers and machine operators). We divided weekly work hours into 47 h and below, 48–52 h, 53 h and above. The European Working Conditions Survey considered that the standard number of work hours per week was 35–47 h [21]; therefore, we considered that working 48 h and above indicated extended work hours. The Korea Labor Standards Act limits extended working hours to 52 h. We divided extended work hours into 48–52 h and ≥ 53 h. Work intensity, job autonomy, and support at work were divided into high and low, and job insecurity was divided into secure and insecure according to the scoring methods by Lu et al. [22].

We explored individual characteristics including gender, age, education, income, job type, cohabitation status, and contribution to household earnings. All subjects were divided into four age groups: < 30, 30–39, 40–49, and ≥ 50 years; and into four groups by educational level: above middle school; middle school graduate, high school graduate, or community college graduate. Average monthly income was divided into intervals of 1,000,000 won (KRW; the Korean currency). Cohabitation status (yes, no); and the contribution made to household earnings (primary earner, secondary earner, or equal earner), were also evaluated.

### Statistical analysis

Work–life balance and work-related and individual characteristics by reference to psychosocial well-being are shown as descriptive statistics. All analyses were carried out using weights. We used the chi-square test to explore the effects of variables on psychosocial well-being. Poisson regression with robust variances was used to determine the estimated prevalence ratios (PRs) for work–life balance and psychosocial well-being (for all samples, and separately for women and men) [23, 24]. Three predictive models were used. Model 1 featured univariate Poisson regression and Models 2 and 3 featured multivariate Poisson regression analysis. Model 2 was adjusted for work-related characteristics (work intensity, weekly working hours, job type, job autonomy, job insecurity, and support at work). Model 3 was adjusted for both work-related and individual characteristics (the covariates of Model 2 plus gender, age, income, cohabitation status, and contribution to household earnings). Variables exhibiting co-linearity were excluded from multivariate analysis. The stratified analysis by women and men did not differ from the results of the overall sample (results not shown). A *p*-value < 0.05 was considered to reflect statistical significance. All analyses were performed with the aid of SPSS (ver. 23.0).

## Results

### Subject distribution and sample characteristics

Table 1 shows data on all subjects. Those with a good work–life balance scored significantly higher in terms of psychosocial well-being. Work intensity did not significantly affect psychosocial well-being. Weekly work for < 47 h, high-level job autonomy, a high support level, job security, and a lower contribution to household earnings, were all associated with significantly better psychosocial well-being. Gender did not significantly affect psychosocial well-being. Age ≤ 39 years improved psychosocial well-being, as did higher education, an income ≥3 million KRW, and a white-collar occupation. A poor work–life balance, work for ≥53 h/week, low-level job autonomy, insecure work, low-level support, older age, and a blue-collar position, were all associated with significantly poorer psychosocial well-being.

**Table 1:** Distribution of sample characteristics with psychosocial well-being

Characteristics	WHO well-being group n (%)		*P* value
	Good well-being	Poor well-being	
Work-life balance			
Good	15,927 (59.1)	11,027 (40.9)	< 0.001
Poor	4075 (46.3)	4735 (53.7)	
Work-related characteristics			
Job type			
White collar	3619 (63.0)	2125 (37.0)	< 0.001
Service	9010 (61.3)	5678 (38.7)	
Blue collar	7536 (48.1)	8131 (51.9)	
Weekly work hours			
≤ 47	13,322 (58.2)	9576 (41.8)	< 0.001
48–52	3699 (56.5)	2849 (43.5)	
≥ 53	3065 (47.6)	3370 (52.4)	
Work intensity			
Low	10,954 (55.7)	8724 (44.3)	0.341
High	9098 (56.2)	7100 (43.8)	
Job autonomy			
high	9502 (58.7)	6672 (41.3)	< 0.001
low	9200 (54.0)	7832 (46.0)	
Job insecurity			
secure	18,344 (56.5)	14,139 (43.5)	< 0.001
insecure	717 (51.4)	678 (48.6)	
Support at work			
high	16,226 (59.4)	11,088 (40.6)	< 0.001
low	2777 (43.1)	3663 (56.9)	
Individual characteristics			
Gender			
Male	10,291 (55.7)	8201 (44.3)	0.404
Female	9887 (56.1)	7741 (43.9)	
Age			
< 30	3098 (62.2)	1882 (37.8)	< 0.001
30–39	5683 (61.7)	3522 (38.3)	
40–49	5840 (56.1)	4564 (43.9)	
≥ 50	5559 (48.2)	5975 (51.8)	
Education			
Below middle school	1726 (39.9)	2599 (60.1)	< 0.001
Graduate high school	6859 (52.1)	6306 (47.9)	
Above college	11,586 (62.2)	7034 (37.8)	
Income (KRW)			
< 1,000,000	2194 (47.6)	2419 (52.4)	< 0.001
1,000,000–1,999,999	6341 (51.6)	5947 (48.4)	
2,000,000–3,000,000	5865 (58.8)	4108 (41.2)	
≥ 3,000,000	5356 (62.9)	3160 (37.1)	
Cohabitation status			
No	2792 (53.6)	2420 (46.4)	< 0.001
Yes	17,386 (56.2)	13,523 (43.8)	
Contribution to household earnings			
Primary earner	11,366 (53.6)	9821 (46.4)	< 0.001
Secondary earner	7281 (60.0)	4863 (40.0)	
Equally earner	1397 (56.9)	1060 (43.1)	

### Association between a poor work–life balance and poor psychosocial well-being

Table 2 presents the multivariate Poisson regression data. All models showed that a poor work–life balance and poor psychosocial well-being were associated. On crude analysis, a poor work–life balance was associated with an increased likelihood (crude PR = 1.32, 95% CI 1.29 to 1.35) of poor psychosocial well-being. After adjusting for work-related characteristics (Model 2), the PR changed slightly (PR = 1.24, 95% CI 1.21 to 1.28). When both individual and work-related characteristics were adjusted (Model 3), the association increased slightly (PR = 1.25, 95% CI 1.21 to 1.28). Model 3 showed that the likelihood of poor psychosocial well-being increased in those working ≥53 h/week (PR = 1.10, 95% CI 1.06 to 1.14); blue-collar status (PR = 1.16, 95% CI 1.11 to 1.21); those with low-level job autonomy (PR = 1.06, 95% CI 1.03 to 1.09); those with low-level support at work (PR = 1.32, 95% CI 1.29 to 1.36); female gender (PR = 1.04, 95% CI 1.01 to 1.07); and increasing age (40–49 years [PR = 1.17, 95% CI 1.12 to 1.23], and ≥ 50 years [PR = 1.21, 95% CI 1.15 to 1.95% CI 26]); and cohabitation (PR = 1.08, 95% CI 1.04 to 1.12). The likelihood of poor well-being was lower for those reporting high-level work intensity (PR = 0.96, 95% CI 0.94 to 0.99); incomes ≥1,000,000–1,999,999 KRW (PR = 0.92, 95% CI 0.87 to 0.96); incomes ≥2,000,000–2,999,999 KRW (PR = 0.82, 95% CI 0.79 to 0.87); incomes ≥3,000,000 KRW (PR = 0.5875, 95% CI 0.71 to 0.79); and those contributing as secondary or equal earners (PR = 0.82, 95% CI 0.79 to 0.85; PR = 0.92, 95% CI 0.87 to 0.97, respectively).

**Table 2:** The association between work-life balance & poor psychosocial well-being in the Korean Working Conditions Survey

	Poor psychosocial well-being		
	PR (95% CI)		
	Model 1a	Model 2b	Model 3c
Characteristics			
Work-life balance			
Good work-life balance	1	1	1
Poor work-life balance	1.32 (1.29 to 1.35)	1.24 (1.21 to 1.28)	1.25 (1.21 to 1.28)
Work-related characteristics			
Job type			
White collar		1	1
Service		1.02 (0.97 to 1.06)	0.99 (0.95 to 1.03)
Blue collar		1.29 (1.23 to 1.34)	1.16 (1.11 to 1.21)
Weekly working hours			
≤ 47		1	1
48–52		0.97 (0.94 to 1.00)	1.00 (0.96 to 1.04)
≥ 53		1.09 (1.05 to 1.13)	1.10 (1.06 to 1.14)
Work intensity			
Low intensity		1	1
High intensity		0.95 (0.93 to 0.98)	0.96 (0.94 to 0.99)
Job autonomy			
High autonomy		1	1
Low autonomy		1.05 (1.02 to 1.08)	1.06 (1.03 to 1.09)
Job insecurity			
Secure		1	1
Insecure		1.02 (0.96 to 1.08)	0.96 (0.90 to 1.02)
Support at work			
High support		1	1
Low support		1.36 (1.33 to 1.40)	1.32 (1.29 to 1.36)
Individual characteristics			
Gender			
Male			1
Female			1.04 (1.01 to 1.07)
Age			
< 30			1
30–39			1.04 (0.99 to 1.09)
40–49			1.17 (1.12 to 1.23)
≥ 50			1.21 (1.15 to 1.26)
Income (KRW)			
< 1,000,000			1
1,000,000–1,999,999			0.92 (0.87 to 0.96)
2,000,000–2,999,999			0.82 (0.79 to 0.87)
≥ 3,000,000			0.75 (0.71 to 0.79)
Cohabitation status			
No			1
Yes			1.08 (1.04 to 1.12)
Contribution to household earnings			
Primary earner			1
Secondary earner			0.82 (0.79 to 0.85)
Equally earner			0.92 (0.87 to 0.97)

*PR* Prevalence ratio, *CI* Confidence interval

a. Crude PR

b. Adjusted for work-related characteristics

c. Adjusted for work-related and individual characteristics

## Discussion

This study explored the association between work–life balance and psychosocial well-being. Previous research has found that work–life balance predicts well-being [7, 9, 10, 14]. Of note, two cross-sectional studies obtained some interesting results. Gröpel and Kuhl [14] revealed that psychosocial well-being is positively correlated with work–life balance (β = 0.40, *p* < 0.001) and negatively correlated with work–family conflict (β = − 0.39, *p* < 0.001) which is an important cause of poor work-life balance. Grant-Vallone and Donaldson [9] found a significant negative association (β = − 0.29, *p* < 0.001) between work–family conflict (an important cause of poor work-life balance) and self-reported well-being. In our study, work–life balance was also associated with psychosocial well-being. The crude analysis revealed an association between poor work–life balance and poor psychosocial well-being (PR = 1.32, 95% CI 1.29 to 1.35). Even after adjusting for work-related and individual characteristics, the well-being of the group with a poor work–life balance was significantly lower (PR = 1.25, 95% CI 1.21 to 1.28).

As mentioned in the introduction the definition of work-life balance is still controversial. Due to this controversy it is important to have a look at the various definitions of work-life balance. Greenhaus, Collins, and shaw defined work-life balance as a balance and equity across multiple roles. Greenhaus, Collins, and Shaw also proposed that work-life balance reflects one’s orientation across different life roles, and inter-role phenomenon. Furthermore, they suggest that work-life balance is the extent to which an individual is engaged in – and equally satisfied with – one’s work role and social role with three components including time balance, involvement balance, and satisfaction balance. [7] Grzywacz & Bass [11] and Frone [25] viewed the psychological part of the work-life balance and defined it as an absence of inter-role conflict and higher levels of inter-role facilitation. Se′necal, Vallerand, and Guay proposed that work-life balance depends on time allocation across various life-roles and a subjective sufficiency of the time available for work and social roles. [6] By adapting the questionnaire from the KWCS ‘In general, do your working hours fit in with your family or social commitments outside work?’ we view and evaluate work-life balance as sufficiency of the time available for work and social roles.

In simple terms, work–life balance must consider multiple aspects of work, family needs, and social life [26]. First, it is necessary to understand why work–life balance affects psychosocial well-being. Role theory and the scarcity hypothesis can be used to examine this [9]. Within role theory, the scarcity hypothesis suggests that individuals have fixed amounts of time and energy for multiple roles [27]. Consequently, increased roles lead to higher role conflict, overload, and negative psychological repercussions. This fixed amount of energy and time results in conflict, stress, and anxiety. Previous studies have supported the notion that multiple roles lead to conflict, overload, and stress and have a negative impact on the well-being and performance of employees [26, 28]. As a result, conflict between work and social life could result in objective and psychological conflict [29]. It can also be explained by needs fulfillment [14]. Well-being is enhanced when goals are met. To reach these goals, resources are required, i.e., time, energy, money, and so forth [30]. The resources available for the goals are thought to be the best predictors of well-being [31]. If one sees time as an important resource, the sufficiency of time to reach that goal can affect well-being. However, not all goals affect well-being. Only goals that satisfy important psychological needs increase well-being [32]. As a result, sufficient time available for work and private life will affect well-being if personal needs are met only within that time [33]. Conversely, insufficient time or conflict within the work and non-work domains may decrease the level of well-being due to needs frustration.

Several important outcomes of poor work–life balance have been documented. Besides decreasing well-being, conflict between work and non-work roles leads to psychological symptoms such as stress, increased depression, anxiety [34], increased somatic complaints [26], and poor physical health [28].

We analyzed data on three predictor models and obtained some interesting results. Our finding that a poor work environment was associated with poor psychosocial well-being is in line with previous results. Long work hours and low-level job autonomy were associated with poor well-being. As also found in previous studies, long work hours and low-level job autonomy mean that workers have poor control over both their work and private lives [35]. An earlier study found that long working hours correlates with higher levels of anxiety and depression [36]. If less time is spent at work, and greater control over work is granted to the worker, psychosocial well-being would improve. Job type, the work environment, and socioeconomic status vary among occupations. We assume that blue-collar workers are more susceptible to poor well-being because of lower incomes, longer working hours, and low job flexibility [37]. However, some authors disagree, arguing that white-collar occupations associated with autonomy and flexibility pose greater job demands and responsibilities that spill over from work into the family, negatively affecting well-being [38]. Poor support at work was also associated with poor well-being. Studies have shown that a low level of support at work can cause problems that spill over into family life, degrading the work–life balance further and compromising psychosocial well-being [39, 40]. In studies conducted in Turkey [41] and Thailand [42], greater work intensity significantly predicted lower psychological well-being. In comparison, our study found a positive association between higher work intensity and good psychosocial well-being. We cannot explain this or cite a relevant prior study regarding this result. We speculate that the two relevant terms used to explore this (“working at a very fast pace” and “working to tight deadlines”) may not have adequately explored the work environment.

Female gender and older age increased the likelihood of poor psychosocial well-being, explained by the fact that both age and gender are associated with the emotional state [43]. Cohabitation status (living with someone) was associated with poor well-being, in line with the results of a previous study; family demands can increase stress that spills over into work [37]. This view is supported by role theory, which suggests that conflict between the increased demands of work and social roles may increase stress-related symptoms and lower psychosocial well-being [27]. According to recent studies, however, cohabitation reduces the likelihood of declining physical health and psychological disorders due to a good combination of work-related and partner roles [44]. A lesser contribution to household income was associated with better well-being, consistent with previous findings [38]. Logically, one would think that the higher the proportion of household income earned the greater the burden on that individual.

Our study had some limitations. First, although we identified an association between a poor work–life balance and poor well-being, the cross-sectional nature of the work means that causal and directional inferences cannot be made. To confirm any directional and causal inferences, a cohort study needs to be conducted. Second, our study used the fourth Korean Working Conditions Survey instead of a customized questionnaire. Considering work–life balance characteristics other than work-related variables is also important. In addition to work domains, family and private social domains and personality traits that might affect psychosocial well-being should also be considered. However, the survey does not contain adequate questionnaire items to analyze family or private social factors, such as cultural traditions and social infrastructure. Third, the variables for work–life balance and psychosocial well-being were dichotomized as good and poor, between which there is ambiguity. Despite these limitations, our study is the first to investigate the association between work–life balance and psychosocial well-being using a large nationwide sample of South Koreans. Although directional inferences are difficult to make due to the cross-sectional data, the possibility of reverse causality remains, as poor psychosocial well-being may potentially increase the likelihood of poor work–life balance. As mentioned above, South Korea ranks very low in terms of the OECD Work–Life Balance Index [13]. It is important to have a close look at work–life balance through various data and studies. In our study, it is meaningful that we used the fourth Korean working conditions survey data, which is representative of South Korea.

## Conclusions

Poor work–life balance was associated with poor psychosocial well-being even after adjusting for some important confounding factors. We expect that further research will identify causal relationships between work–life balance and psychosocial well-being. Furthermore, our findings, combined with the current situation in South Korea, suggest that it is necessary to implement measures assisting workers to balance their work with their private lives, thus improving well-being.

## Abbreviations

CI: Confidence Interval
KWCS: Korean Working Conditions Survey
PR: Prevalence Ratio
WHO: World Health Organization
